# Two-, three-, and four-person mixtures in forensic casework: difficulties and questions

**DOI:** 10.3325/cmj.2011.52.653

**Published:** 2011-10

**Authors:** Allan Jamieson, Scott Bader, Georgina Meakin, Carrie Mullen

**Affiliations:** The Forensic Institute, Glasgow, United Kingdom

**To the Editor:** The article by Perez et al (1) ostensibly provides “guidelines to estimate the number of contributors to two-, three-, and four-person mixtures.” Unfortunately, the article fails to provide sufficient information on the authors’ interpretation process and thus any reliable support for the proposed guidelines.

The interpretation of DNA profiles in forensic work is required to assist in the identification of the possible sources (number and identity) of a crimestain. Notwithstanding the difficulties in interpreting low-level artifacts such as stutter and the increased difficulty involved when using low template methods in routine casework to reliably identify an allele, often the only known facts from a profile are the total amount of DNA and the type and number of alleles at each locus. Even these are frequently either unknown or uncertain in low-template DNA work.

Although there may be reasons to identify numbers of contributors greater than three, it is difficult to see the practical application of such because of the difficulties with the statistical analysis to establish the weight of any evidence of individualization from such profiles.

## Reproducibility

It is obscure how these experiments could be replicated by reference to the article, thus failing to satisfy the normal scientific requirement of enabling others to reproduce the experiments. The lack of clarity makes it impossible to assess the validity of the inferences from the results. No key parameter (ratio of contributors, total amount of DNA, number of contributors, ethnic composition) is sufficiently documented to enable a sensible appraisal of the data, and certainly not to assess whether the proposed guidelines are supported. There is no complete list of both ratios and amounts of DNA used. Each key parameter appears to have been consolidated in groups. It is also obscure what rationale produced the numbers of samples listed under “Amplification” (p.317), and impossible to know the amount of DNA at any ratio.

Although the work is intended to examine the effect of different numbers of contributors and different ratios of the contributors’ DNA, the data do not permit assessment of these.

## Data analysis

The data are simply insufficient to enable any sensible assessment of the support they provide for the proposed guidelines.

It is likely that much of the data may be based on false assumptions caused by a flawed protocol that fails to provide accurate estimates for the amount of DNA and/or contributors to a purposeful mixture. This is because of the basic setup protocol: It is implied (“Quantification”) that the method used to create different initial amounts (and ratios) of DNA was dilution of a sample of known quantity. However, it is known that, especially at the lower levels being used in this work, stochastic effects will cause significant differences between the expected amount of DNA (based on dilution) and the actual amount of DNA. For example, in the 25pg 5:1:1 mixture the lower contributors to this mixture are expected to have 25/7 = 3.6pg DNA. Less than a single cell’s expected content. It is therefore not surprising that these exhibited significant dropout (stochastic variation), although the extent of that dropout is not documented and this further hampers assessment. However, in a casework sample the analyst would not have the luxury of knowing the number of contributors – the very question that the guidelines seek to answer.

The authors curiously argue that three-person mixtures that appear to be two-person mixtures should be considered two-person mixtures. It is not obvious what they mean by such phrases as “better described as” and “probably best described as.” Nor is it obvious how this would be helpful in casework since in casework the expert is trying to decide the number of contributors to the profile, rather than having that as a known fact. Do the authors mean that, in casework such samples would be considered for statistical analysis as 2-person mixtures, or that they should be considered as 2-person mixtures for the purpose of these experiments? If the former is true, then the interpretation would be simply wrong, and if the latter is true it has the appearance of selecting the data to fit a preconceived hypothesis. It is not stated how many samples produced inconclusive results and have therefore been removed from the data set.

We suggest that the correct approach to this type of work is to perform the analyses and then determine, on the basis of the observed results, what guidelines (if any) can be derived. It is therefore a source of concern that the data used to derive the guidelines appears to have been subject to “editing” on the basis of data selection (those with the greatest number of labeled alleles, according to the “Data Collection” section), which is only possible knowing the actual contributors or having the facility for multiple runs; a circumstance not always available in casework. We argue the same regarding the use of software data filters also described in the said section.

## Applicability

Although the authors claim that the guidelines were, “useful tools to distinguish low template and high template two-, three-, and four-person mixtures,” at no point do they appear to have tested that proposition using blind trials. Such blind trials would have forced the analyst to make decisions using the guidelines, which would, on the basis of the results reported in this paper, derive erroneous attributions of the number of contributors.

## Conclusion

In summary, this paper cannot be regarded as sufficient to support the guidelines that the authors propose. For that reason, it is not necessary to address here the substantial scientific problems associated with the additional work on touched objects contained in the paper.
